# Retraction: Application of artificial bee colony algorithm based on homogenization mapping and collaborative acquisition control in network communication security

**DOI:** 10.1371/journal.pone.0345997

**Published:** 2026-04-08

**Authors:** The PLOS One Editors

The *PLOS One* Editors retract this article [1] due to concerns about potential manipulation of the publication process. These concerns call into question the validity and provenance of the reported results. We regret that the issues were not identified prior to the article’s publication.

JZ and XW did not agree with the retraction. HW either did not respond directly or could not be reached.
